# Improved Genome Sequence of Australian Methicillin-Resistant Staphylococcus aureus Strain JKD6159

**DOI:** 10.1128/mra.01129-22

**Published:** 2023-01-18

**Authors:** Ryan R. Wick, Louise M. Judd, Ian R. Monk, Torsten Seemann, Timothy P. Stinear

**Affiliations:** a Department of Infectious Diseases, Central Clinical School, Monash University, Melbourne, Australia; b Department of Microbiology and Immunology, University of Melbourne at the Peter Doherty Institute for Infection and Immunity, Melbourne, Australia; Indiana University, Bloomington

## Abstract

Staphylococcus aureus strain JKD6159 represents a prominent community-acquired methicillin-resistant S. aureus (MRSA) clone in Australia. Here, we report an improved assembly of the original S. aureus JKD6159 genome sequence. By using deep sequencing with multiple technologies combined with carefully curated assembly and polishing, we believe the assembly to contain zero errors.

## ANNOUNCEMENT

Staphylococcus aureus strain JKD6159 is a methicillin-resistant clone of S. aureus (1) belonging to the sequence type 93 (ST93) lineage, which was first reported in Australia but is also found in Europe and New Zealand (2). This strain was isolated in Australia in 2004 from a patient in whom it caused septicemia and multifocal abscesses (3). While its complete genome sequence was originally published in 2010 (1) (NCBI Assembly accession no. GCF_000144955.1), we have since resequenced and reassembled JKD6159 using modern platforms and bioinformatic tools to produce a genome sequence, which we believe to be free of errors.

The isolate was cultured overnight at 37°C (200 rpm) in Bacto Brain Heart Infusion broth (Becton Dickinson), and DNA was extracted using GenFind V3 according to the manufacturer’s instructions (Beckman Coulter) using lysozyme and proteinase K without size selection. We generated 1,831,719 reads (5.59 Gbp, *N*_50_ of 4.2 kbp) using an R10.4 MinION flow cell by using the SQK-NBD112.96 kit. The reads were basecalled and adapter trimmed with Guppy v6.1.7 (dna_r10.4_e8.1_sup model). We performed quality control (QC) by discarding reads <6 kbp and the worst 10% of reads using Filtlong v0.2.0 (4), resulting in 135,671 reads (1.82 Gbp, *N*_50_ of 15.2 kbp). We also generated 6,844,242 paired-end 150-bp reads (998 Mbp) on an Illumina NextSeq 500 using a Nextera XT preparation. Illumina QC was performed using fastp v0.23.2 (5) with default parameters.

We assembled the long reads using Trycycler v0.5.3, following the “extra-thorough” instructions in Trycycler’s documentation (using Canu v2.3 [6], Flye v2.9 [7], miniasm v0.3/Minipolish v0.1.3 [8, 9], NECAT v20200803 [10], NextDenovo v2.5.0/NextPolish v1.4.0 [11, 12], and Raven v1.8.1 [13]). This produced three circular contigs, which were a 2,818,668-bp chromosome, a 43,131-bp phage, and a 20,730-bp plasmid. We then ran Medaka v1.6.0 (14), which made 19 single base pair changes to the chromosome and no changes to the phage or plasmid. Short-read polishing with Polypolish v0.5.0 (15) made 26 single base pair changes to the chromosome and no changes to the phage or plasmid. We then ran POLCA v4.0.9 (16), which made no changes, followed by FMLRC2 v0.1.7 (17), which changed seven regions of the chromosome, but each was manually assessed in Integrative Genomics Viewer (IGV) v2.13.0 (18), determined to be an introduced error, and rejected. For all tools, default parameters were used except where otherwise noted.

The circular phage sequence was identical to an integrated phage in the chromosome. To verify that there were no differences between the circular and integrated phage sequences, we produced a 100× Oxford Nanopore Technologies (ONT) read set with Filtlong v0.2.0, which was 7,630 reads with an *N*_50_ of 40.6 kbp (long enough for most reads to uniquely align). We then repeated the entire assembly/polishing process, which produced an identical result to our previous assembly. Since the integrated and circular phage sequences were confirmed to be identical, we removed the redundant circular phage. To verify that no small plasmids were excluded, we performed a short-read-first hybrid assembly using Unicycler v0.5.0 (19) but did not find any additional plasmids. Our final assembly had a 2,818,670-bp chromosome and a 20,730-bp plasmid (pSaa6159) with 32.8% GC content. After annotation with the NCBI Prokaryotic Genome Annotation Pipeline (PGAP) v6.2, the chromosome contained 2,701 coding sequences, 59 tRNAs, 19 rRNAs, 3 noncoding RNAs (ncRNAs), and 1 transfer-messenger RNA (tmRNA), and pSaa6159 contained 24 coding sequences.

To verify the assembly’s accuracy, we produced R9.4.1 MinION reads (SQK-RBK110.96 kit; 255,545 reads, 2.11 Gbp, *N*_50_ of 22.4 kbp, generated from the same DNA) and repeated the process with Trycycler v0.5.3, Medaka v1.6.0 (181 changes), Polypolish v0.5.0 (49 changes), and POLCA v4.0.9 (one change verified in IGV), and the result was identical to our R10.4-plus-Illumina assembly. Finally, we assembled the S. aureus JKD6159 genome using previously sequenced PacBio RS II reads (20) (628,002 reads, 797 Mbp, *N*_50_ of 2.4 kbp) with Trycycler v0.5.3 and Quiver v2.3.3 (21) (24 changes), and the result was also identical. The fact that three alternative approaches (R10.4-plus-Illumina, R9.4.1-plus-Illumina, and PacBio RS II) had no discrepancies supports our claim that this S. aureus JKD6159 assembly contains zero errors.

### Data availability.

The revised genome sequence for S. aureus JKD6159 has been deposited in GenBank with accession number GCF_000144955.2. Sequencing data are available on SRA (Illumina, accession number SRR21386014; ONT R10.4 raw, accession number SRR21386013; ONT R10.4 basecalled, accession number SRR21386012; ONT R9.4.1 raw, accession number SRR21386011; ONT R9.4.1 basecalled, accession number SRR21386010; PacBio RS II raw, accession number ERR1213694; and PacBio RS II basecalled, accession number SRR21386009) and figshare (https://bridges.monash.edu/articles/dataset/S_aureus_JKD6159_sequencing_data/21007033).
